# Immortalization of MEF is characterized by the deregulation of specific miRNAs with potential tumor suppressor activity

**DOI:** 10.18632/aging.100353

**Published:** 2011-07-11

**Authors:** Milena Rizzo, Monica Evangelista, Marcella Simili, Laura Mariani, Letizia Pitto, Giuseppe Rainaldi

**Affiliations:** ^1^Laboratory of Gene and Molecular Therapy, Institute of Clinical Physiology, CNR, Pisa, Italy;; ^2^ Istituto Toscano Tumori, Firenze, Italy

**Keywords:** mouse embryo fibroblasts, immortalization, microRNAs, genome instability

## Abstract

The life span (Hayflick limit) of primary mouse embryo fibroblasts (MEF) in culture is variable but it is still unclear if the escape of the Hayflick limit is also variable. To address this point MEF were expanded every fifteen days (6T15) instead of every three days (6T3) until they became immortal. With this protocol MEF lifespan was extended and immortalization accordingly delayed. By testing a panel of genes (p19ARF, p16, p21) and miRNAs (miR-20a, miR-21, miR-28, miR-290) related to primary MEF senescence, a switch of p21 from up to down regulation, the down regulation of specific miRNAs as well as a massive shift from diploidy to hyperdiploidy were observed in coincidence with the resumption of cell proliferation. Collectively, these data indicate that the inactivation of genes and miRNAs, important in controlling cell proliferation, might be determinant for the escape from the Hayflick limit. In support of this hypothesis was the finding that some of the down regulated miRNAs transfected in immortalized MEF inhibited cell proliferation thus displaying a tumor suppressor-like activity.

## INTRODUCTION

The Hayflick limit of primary mouse embryo fibroblasts (MEF) is variable since the growth conditions can either reduce or extend it [1-3]. We have reported that primary MEF expanded every three days (6T3 protocol) underwent four population cell doubling and thereafter cell proliferation was fully abolished and SA-β-gal^+^ cells induced [4]. However it is known that MEF senescence can be induced prematurely, before the end of the physiological lifespan, by cellular stresses such as the over expression [5] or down regulation [6] of single oncogenes and DNA damaging drugs [7-9]. Recently, it has been reported that miRNAs are involved in senescence of MEF as well as of human diploid fibroblasts [10,11].

An unsolved question is which cells are able to escape the *in vitro* lifespan limit. So far, the molecular characterization of immortalized MEF cell lines has shown that functional silencing of either the *INK4a/ARF* locus [12] or p53 [13] appears necessary to bypass senescence. A recurrent concept is that mutational events occurring in culture have a key role. If mutations confer a proliferative advantage, mutated cells can bypass the *in vitro* lifespan limit and rapidly replace the existing population [14,15]. According to this view, the time spent in culture should affect immortalization. We modified the 6T3 expansion protocol of primary MEF [4] by lengthening the interval between passages (15 days versus 3 days) and reiterating it until MEF became immortal. Here we report that: i) the life span was extended up to 8 population cell doubling and immortalization was consequentially delayed; ii) p21 down regulation marks the switch from primary to immortalized MEF; iii) deregulation of genes and miRNAs which control cell proliferation pathways correlate with the immortalization process; iv) down regulated miRNAs can behave as tumor suppressors.

## RESULTS

### 1. The pro senescence axis p53/p21 is disrupted in immortalized MEF

**Figure 1:**
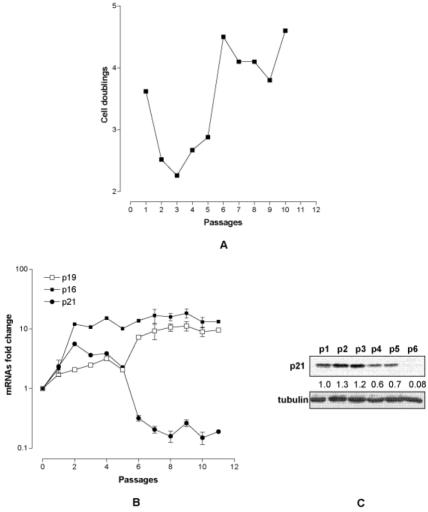
Characterization of MEF under 6T15 propagation regimen. (A) Proliferation curve of MEF expressed as cell doublings per passage. (B) Quantification of *p19ARF*, *p16* and *p21* transcripts per passage (each point represent one passage) normalized to that of MEF at passage 0. (C) p21 protein level at the various passages. The value reported under each lane represents the average of two independent experiments.

To investigate the relative importance of the time in culture versus the number of population cell doubling in the MEF immortalization process we modified the split time. MEF p0 (passage 0) were thawed, and expanded to obtain MEF p1. Thereafter MEF p1 were collected, diluted at the appropriate concentration (6x10^5^) and grown for 15 days with a medium change every three days (6T15 protocol), before the next trypsinization and expansion. The 6T15 protocol was reiterated 10 times for a total of 150 days in culture. During the first three passages a reduced number of population cell doubling was observed; afterwards a population of proliferating MEF emerged, which rapidly increased (p5) and replaced the preexisting cell population (p10) (Fig. 1A). At the molecular level we found that both p19ARF and p16 were progressively up regulated during passages (with respect to the spontaneous level of MEF p0) (Fig. 1B). Vice versa the expression of p21 was biphasic: while it was up regulated till p3 it decreased thereafter with a clear switch toward under expression at p6 (Fig. 1B,C). As p21 is under the direct control of p53, a disruption of the p53/p21 axis can be hypothesized at the basis of immortalization.

### 2. Genome stability and miRNA signatures are modified in immortalized MEF

**Figure 2:**
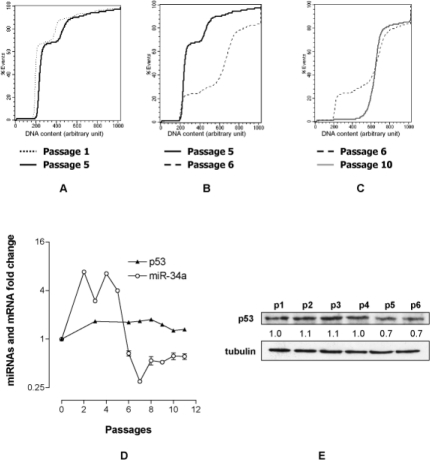
MEF immortalization is characterized by a p53-dependent events. DNA content profile per cell at p1/p5 (A), p5/p6 (B) and p6/p10 (C). (D) Fold change of miR-34a and p53 transcripts at various passages normalized to that of MEF at passage 0. (E) p53 protein level at the various passages. The value reported under each lane represents the average of two independent experiments.

The down regulation of p21 strongly suggests a loss of p53 activity. In accordance with the hypothesis, FACS analysis showed that while the DNA content distribution curve per cell of MEF p1 and MEF p5 were very similar and typical of diploid cells (Fig. 2A) a massive shift toward cells with higher DNA contents was observed in MEF p6 (Fig. 2B). Hyperdiploid cells, which emerged from diploid primary MEF, are indicative that genomic stability, controlled by p53 [16-18], is lost. It is of note that a stable hyperdiploid cell population is selected within four passages (p6-p10) (Fig. 2C). To strengthen the hypothesis of a functional loss of p53, in parallel we determined the expression profiles of p53 and of miR-34a directly controlled by p53. We found that p53 mRNA (Fig. 2D) and protein levels did not significantly change up to p6 (Fig. 2E); conversely miR-34a appears to increase till p4, starts to decrease at p5 to become markedly down regulated at p6 (Fig. 2D) reinforcing the idea that p53 becomes non-functional.

### 3. The senescence related miRNAs are down regulated in immortalized MEF

**Figure 3:**
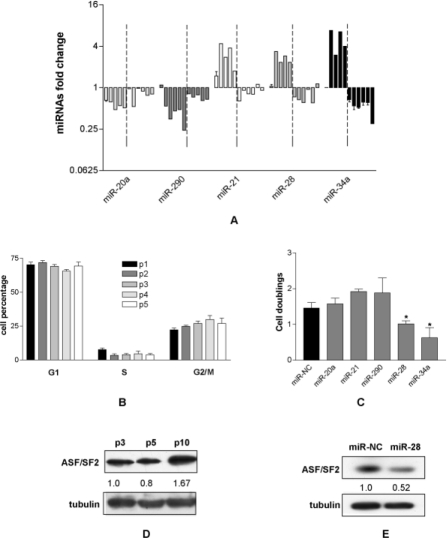
The re-expression of miRNAs down regulated in immortalized MEF reduces cell proliferation. (A) Quantification of miR-20a, miR-21, miR-28, miR-290 and miR-34a per passage normalized to that of MEF at passage 0. Dashed lines indicate the transition from passage 5 to 6. (B) Cell cycle phase distribution (%) of MEF from p1 to p5. (C) Population cell doubling of immortalized MEF after transfection of miR-NC, miR-20a, miR-21, miR-28, miR-290 and miR-34a. Each bar represents the mean ± SD of three biological replicates (*=p<0.05). (D) Expression of ASF/SF2 protein in primary (p3 and p5) and immortalized (p10) MEF. The value reported under each lane represents the average of two independent experiments. (E) Expression of ASF/SF2 after the transfection of immortalized MEF with either miR-NC or miR-28. The value reported under each lane represents the average of two independent experiments.

As the p53/p21 axis of primary MEF was disrupted in immortalized MEF, we investigated whether the time course of the signatures of miRNAs related to either premature (miR-20a [19]) or replicative (miR-21, miR-28 [20], miR-290 [4]) primary MEF senescence were also modified. The analysis showed that while miR-20a and miR-290 were down regulated till p6 (Fig 3A) miR-21 and miR-28 were up regulated. It is worth noting that the up regulation of miR-21 and miR-28 is in agreement with findings in MEF replicative senescence, while miR-290 down regulation is the opposite of previous observations because we have shown that miR-290 steadily increased when either spontaneous or nocodazole-induced MEF G1 blocked tetraploid cells were present [4]. For that, we examined the cell cycle of p1-p5 MEF. We found that under 6T15 protocol no significant accumulation of G2/M cells was observed (Fig. 3B) suggesting that the failed accumulation of G2/M cells was responsible for the lack of miR-290 up regulation. The miRNA signatures changed markedly after p6: while miR-20a and miR-290 remain down regulated, although to a lesser extent, miR-21 and miR-28 switched from up to down regulation (Fig. 3A). These data suggest that the deregulation of these two senescence related miRNAs, besides miR-34a, is involved in MEF immortalization.

### 4. The transfection of the down regulated miRNAs reduces the proliferation of immortalized MEF

To investigate if the deregulated miRNAs play an active role in the immortalization, immortalized MEF (p10) were transfected with miR-21, miR-28 and miR-34. In addition we tested miR-20a and miR-290 whose expression was not affected by the immortalization. The proliferation data showed that miR-20a and miR-290 did not affect cell proliferation as expected, the down regulated miR-28 and miR-34 significantly reduced the proliferation of immortalized MEF with similar efficiency, while miR-21 did not inhibit cell proliferation (Fig. 3C). These data indicate that the re-expression of miR-34a and miR-28 (and consequently of their controlled pathways) was sufficient to reduce the proliferative rate of immortalized MEF and suggest that miR-28, like the tumor suppressor miR-34a, could have a tumor suppressor activity.

### 5. MiR-28 behaves as tumor suppressor miRNA

It is well known that miR-34a has a widespread tumor suppressor activity [21,22]. We show that it behaved as such in immortalized MEF as its re-expression inhibits cell proliferation, probably by partially restoring the p53 pathway. Interestingly miR-28, as far as we know, is not directly linked to the p53 controlled pathways. We have previously reported that in primary MEF miR-28 targets the proto-oncogenic splicing factor ASF/SF2 and that in turn miR-28 over-expression induces both apoptosis and senescence of primary MEF via ASF/SF2 down regulation [20]. This prompted us to verify the expression of ASF/SF2 in primary and immortalized MEF. As expected the expression of ASF/SF2 was higher in immortalized than in primary MEF (Fig. 3D) suggesting a possible post transcriptional control of miR-28 on ASF/SF2 expression. To confirm the hypothesis, we transfected immortalized MEF (p10) with miR-28 and found 50% reduction of ASF/SF2 expression (Fig. 3E), suggesting that miR-28 control cell proliferation by targeting ASF/SF2. These results strengthen the idea that miR-28 has a tumor suppressor-like activity and might be suitable to be tested in tumor cell lines defective in miR-28 content.

## DISCUSSION

The *in vitro* life span defines the number of population cell doubling which primary cells can undergo in culture. We have reported that MEF under the 6T3 expansion regimen were able to undergo four population cell doubling before achieving senescence. In this work we reported that by modifying the propagation regimen and growth conditions (6T15 protocol) MEF slowed down without reaching a complete cell proliferation block and restarted proliferation after about eight population cell doubling (passage 3). These results suggest that activation of p53 with the consequent induction of p21 in this case may favor proliferation arrest rather than senescence as previously described [23]. Interestingly the *in vitro* life span of MEF was extended both in terms of population cell doubling and in terms of days in culture, in keeping with the hypothesis that p53 activation, by inducing quiescence rather than senescence may increase the cell life span [24]. The immortalization process is delayed accordingly, suggesting that it does not strictly depend on the number of cell doubling or the time that the cells spend in culture (45 culture days in this case).

The spontaneous immortalization of primary MEF has been attributed to either the inactivation of p53 or the loss of the *INK4a/ARF* locus (p19ARF, p16) [25], two loci strictly related to cell proliferation. In this work we found that both loci were transcribed and translated during passages whereas p21 shifted from up to down regulation after p5. As p21 expression is mostly p53 dependent we argued that despite p53 continued to be expressed, it may not be functional any more. In support of this conclusion are the down regulation of the p53 dependent miR-34a and the loss of genomic stability revealed by the massive appearance of hyperdiploid cells [16-18]

A recurrent concept is that immortalization is due to mutations induced by DNA damage occurring during the population expansion [14]. The loss of p53 activity found in immortalized MEF could be due to either point mutation or loss of one allele which appears to be sufficient to alter cell growth [15]. In any case the sharp increase of cell proliferation suggests that the immortalization process is caused by the concomitant alteration of multiple pathways. We found that the expression of miR-20a, miR-21, miR-28 and miR-290, all involved in MEF senescence, were deregulated in coincidence with p21 down regulation and increase of cell proliferation. As a high number of genes are potentially targeted by one miRNA [26], we argued that the deregulation of these miRNAs could be in part responsible for the absence of cell proliferation control characteristic of immortal cells. In particular we demonstrate that by replacing miR-28 and miR-34a, under expressed in immortalized MEF, cell proliferation was reduced suggesting that both miRNAs are implicated in immortalization. Evidence in support of an anti proliferative role of miR-28 is already available. We have shown in primary MEF that miR-28 targets the proto-oncogene ASF/SF2, a splicing factor involved in the alternative splicing of many transcripts [27,28] and its over expression induces apoptosis and senescence by down regulating ASF/SF2 [20]. Our results demonstrate that also in immortalized MEF miR-28 targets ASF/SF2 suggesting that its tumor suppressor activity is due to ASF/SF2 inhibition, although other targets cannot be excluded.

The fact that the replacement of miR-28 in immortalized MEF reduced cell proliferation to the same extent as miR-34a further strengthens the hypothesis that miR-28 represents a novel TS miRNA. MiR-34a is a well known TS miRNA able to inhibit cell proliferation of a wide range of tumor cells [21,29-31] as well as of human fibroblasts which extend their replicative capacity when treated with miR-34a antisense [32]. Conversely miR-290 and miR-20a involved in culture and stress induced senescence of primary MEF were not able to inhibit proliferation of immortal MEF in keeping with the idea that miRNAs behave differently in different cellular context [33]. In conclusion the delayed immortalization obtained with this protocol implies that point mutations; due to DNA damage accumulated during population expansion [14], are not the only molecular events at the basis of immortalization. The most notable feature which marks the immortalization process is the drastic p21 down regulation possibly due to p53 functional inactivation. Associated to the sudden down regulation of p21 was the induction of hyperploidy, suggesting that genomic instability and/or epigenetic changes are also responsible for the immortalization process. Interestingly, the switch of p21 expression was accompanied by the change of the signature of miRNAs related to MEF senescence, including the p53-dependent miR-34 and miR-28 [20]. In particular, the down regulated miR-28, behaved as a TS miRNA, when transfected in immortalized MEF, analogously to the well known TS miR-34a, indicating that the comparison of the miRNA signature of primary versus immortalized cells could allow the identification of novel miRNAs with potential tumor suppressor-like activity.

## MATERIALS AND METHODS

### Reagents

miRNeasy mini kit, QuantiTect Reverse Trascription Kit, miScript Reverse Transcription Kit, miScript SYBR Green PCR Kit (QIAGEN, Milano, Italy); Dulbecco's Modified Eagle Medium-High Glucose (D-MEM-HG), foetal bovine serum (FBS) (Invitrogen, CA, USA); LightCycler 480 Probes Master, Universal ProbeLibrary LNA Probes; LightCycler 480 SYBR Green I Master (Roche Diagnostic, Mannheim, Germany); X-Gal (5-bromo-4-chloro-3-indolylb-D-galactoside); propidium iodide, anti-α-tubulin (Sigma-Aldrich Corporation, Missouri, USA); anti-p21 (Santa Cruz Biotechnology, Inc.); ECL, Hybond-C extra membranes (Amersham); anti-p53 (Cell Signaling Technology). The anti-ASF/SF2 was a gift of Dr. Adrian Krainer. MiR-20a, miR-21, miR-28, miR-34, miR-290 and miR-NC (negative control) (GenePharma Shanghai, China)

### Cells and culture conditions

MEF were isolated from 13.5d mouse embryos, expanded and then replated every three days (6T3 protocol). For 6T15 protocol MEF p1 were collected, diluted at the appropriate concentration (6x10^5^) and grown for 15 days with a medium change every three days, before the next trypsinization and expansion. MEF were grown in Dulbecco's Modified Eagle Medium-high glucose (DMEM-HG)-10% FBS at 37°C in a humidified atmosphere containing 6% CO_2_.

### Cell proliferation

Cell proliferation was measured as number of population cell doubling per passage (CD= ln(N_f_/N_i_)/ln2) where N_f_ is the final number of collected cells (day 3) and N_i_ the initial number of seeded cells (day 0).

### MiRNAs transfection

Immortalized MEF (p10) were seeded at cell density of 1.0x10^5^ per 30 mm diameter dish. After 24 hours cells were transfected with either miRNAs under test or a double-stranded oligonucleotide, named miR-NC. Briefly, 15 μl Optimem and 25 μl transfection buffer plus 80 nM miRNA were mixed with a solution of Gene Silencer (5 μl) plus Optimem (25 μl). After 15 minutes incubation, Optimem was added up to 800 μl. After 6 hours the medium was replaced with complete DMEM.

### Quantification of miRNAs and genes with Q-Real-time PCR

Total RNA was extracted from 1x10^6^ cells using the miRNeasy mini kit (Qiagen) following the manufacturer's recommendations. To quantify p19ARF, p16 and p21 transcripts, 1μg of total RNA was reverse transcribed using QuantiTect Reverse Trascription Kit (Qiagen). Real-time PCR (qRT-PCR) was carried out with LightCycler 480 (Roche) using LightCycler 480 SYBER Green I Master (Roche). Mature miR-20a, miR-21, miR-28, miR-34a and miR-290 were quantified using the miScript System: 1μg of total RNA was retrotranscribed with miScript Reverse Transcription Kit (Qiagen) and qRT-PCR was carried out using miScript SYBR Green PCR Kit (Qiagen). All reactions were performed in triplicate. Relative quantification of gene expression was calculated with the fit point method. Transcript values were normalized with those obtained from the amplification of the internal controls (GAPDH for transcripts and U6 for miRNAs). The following oligonucleotides were used: p19ARF, forward (F) (5'-CATGGGTCGCAGGTTCTTG-3') and reverse (R) (5'-GCTCGCTGTCCTGG GTCTC-3'); p16, F (5'-CGACGGGCATAGCTTCAG-3') and R (5'-GCTCTGCTCTTGGGATTGG-3'); p21, F (5'-TCCACAGCGATATCCAGACA-3') and R (5'-GGACATCACCAGGATTGGAC-3'); p53, F (5'-ATGCCCATGCTACAGAGGAG-3') and R (5'-AGACTGGCCCTTCTTGGTCT-3'); GAPDH, F (5'-GCCTTCCGTGTTCCTACCC-3'), R (5'-TGCCTGCTTCACCACCTTC-3'); miR-20a, F (5'-TAAAGTGCTTATAGTGCAGGTAG-3'); miR-21, F (5'-TAGCTTATCAGACTGATGTTGA-3'), miR-28, F (5'-AAGGAGCTCACAGTCTATTGAG-3'); miR-34a, F (5'-TGGCAGTGT CTTAGCTGGTTGT-3'); miR-290, F (5'-gctaatcttctctgtatcgttccaa-3'); U6, F (5'-CGCAAGGATGACACGCAAATTC-3').

### Western Blot analysis

Equivalent amounts of proteins were resolved on 10% SDS-PAGE gels and transferred to Hybond-C extra membranes by electro blotting. The resulting blots were blocked with 5% nonfat dry milk solution. Anti-α-tubulin (1:1000), anti-p53 (1:500), anti-p21 (1:1000), anti-ASF/SF2 (1:2000) were used. Incubation was performed overnight at 4°C and bands were revealed after incubation with the recommended secondary antibody coupled to peroxidase using ECL. Scanned images were quantified using scion Image software and normalized to α-tubulin.

### Cell cycle

Samples of 5x10^5^ of cells under test were fixed with 95% ethanol, stained with 50 μg/ml propidium iodide (PI), incubated over night at 4°C and cell cycle analyzed using a FACScalibur cytofluorimeter. The Kolmogorov-Smirnov Statistic was used to represent the DNA content profile of MEF at the various passages.

### Statistical analysis

Data were analyzed using GraphPad Prism (GraphPad Sofware, Inc., San Diego, CA). Comparisons were evaluated by unpaired *t*-test. A value of p<0.05 (*) was considered statistically significant.
